# Collecting Self-reported Physical Activity and Posture Data Using Audio-based Ecological Momentary Assessment

**DOI:** 10.1145/3678584

**Published:** 2024-09-09

**Authors:** HA LE, RITHIKA LAKSHMINARAYANAN, JIXIN LI, VARUN MISHRA, STEPHEN INTILLE

**Affiliations:** Khoury College of Computer Sciences, Northeastern University, USA; Khoury College of Computer Sciences and Bouvé College of Health Sciences, Northeastern University, USA; Khoury College of Computer Sciences and Bouvé College of Health Sciences, Northeastern University, USA; Khoury College of Computer Sciences and Bouvé College of Health Sciences, Northeastern University, USA; Khoury College of Computer Sciences and Bouvé College of Health Sciences, Northeastern University, Boston, MA

**Keywords:** Ecological Momentary Assessment, Experience Sampling, Physical Activity Measurement, Ubiquitous and Wearable Computing, Audio, Microinteraction

## Abstract

*μ*EMA is a data collection method that prompts research participants with quick, answer-at-a-glance, single-multiple-choice self-report behavioral questions, thus enabling high-temporal-density self-report of up to four times per hour when implemented on a smartwatch. However, due to the small watch screen, *μ*EMA is better used to select among 2 to 5 multiple-choice answers versus allowing the collection of open-ended responses. We introduce an alternative and novel form of micro-interaction self-report using speech input – audio-*μ*EMA– where a short beep or vibration cues participants to verbally report their behavioral states, allowing for open-ended, *temporally dense* self-reports. We conducted a one-hour usability study followed by a within-subject, 6–day to 21-day free-living feasibility study in which participants self-reported their physical activities and postures once every 2 to 5 minutes. We qualitatively explored the usability of the system and identified factors impacting the response rates of this data collection method. Despite being interrupted 12 to 20 times per hour, participants in the free-living study were highly engaged with the system, with an average response rate of 67.7% for audio-*μ*EMA for up to 14 days. We discuss the factors that impacted feasibility; some implementation, methodological, and participant challenges we observed; and important considerations relevant to deploying audio-*μ*EMA in real-time activity recognition systems.

## 1 INTRODUCTION

Continuous measurement of human behaviors in naturalistic settings is an important research topic in fields such as human-computer interaction, ubiquitous computing, and health sciences. Data collected in free-living situations can be used to measure and model behavior and to build new just-in-time interventions. Systems that capture in-situ behavioral data may prove valuable when creating real-time, personalized models and interventions that are tuned to individual behavioral patterns.

For example, accurate measurement of activity and posture could enable new health and wellness applications and patient care assistive tools. However, researchers often require substantial, continuous, *temporally dense* labeled data, not only to develop new activity and posture detection algorithms, but also to validate and benchmark existing state-of-the-art approaches. One common option is to collect activity and posture datasets in controlled settings [7]. Activities can be performed under staff observation and thus carefully labeled, but the set of activities observed may not reflect the scope and complexity of activity in daily life. Another option is to use a first-person (egocentric) camera to record behavior that staff can subsequently label [30, 39]. However, a first-person camera can be inconvenient for participants and those around them; it may also induce measurement bias as people change their lifestyles to avoid the camera capturing personal or sensitive information. Moreover, on-body cameras available today are either bulky, uncomfortable, or have poor battery capacity when continuously recording and cannot last a full waking day.

A more practical approach is to prompt participants to complete a self-report survey on a phone using ecological momentary assessment (EMA) surveys or via a recall diary prompted at the end of the day. EMA surveys and recall diaries have limitations as well. First, they create a response burden because study participants need to physically pull out their devices and answer the surveys. After-the-fact surveys [69] suffer from recall biases because of the limits of human memory. EMA uses momentary responses to minimize recall bias, but it introduces more interruption and response burden. Given the burden, both self-report methods are rarely prompted more than once every hour, resulting in a lack of temporal density; moreover, this prompting rate is ill-suited for precisely annotating the start/stop transition times between activities. This lack of annotation precision can decrease the performance of an activity prediction model that needs momentary labels and periods of time for training. *μ*EMA (micro-EMA) is a specific type of EMA where answer-at-a-glance multiple-choice self-report questions (e.g., “Are you eating?” with answer options “Yes” and “No”) are prompted at a high temporal density; the method has been deployed longitudinally with prompting rates as high as four times per hour [59]. When considering its use for generating activity labels, *μ*EMA may be best suited to confirm a prediction where answers are limited to 2 to 5 options; the method does not allow for at-a-glance capture of open-ended responses, selecting from a long list of options, or answering multiple questions (e.g., asking about activity and then posture).

In this paper, we explore a new method of collecting behavioral labels *in-the-wild* using a voice-input *μ*EMA system: audio-*μ*EMA. Audio-*μ*EMA may be useful for tasks where temporally dense gold-standard self-reported labeling is required while someone is engaged in complex, everyday tasks. We explored two device modalities for the audio-*μ*EMA system: participants could either use an earable device or a smartwatch. By cueing participants to report their activity and posture data verbally, our system was able to collect activity and posture labels every 2 to 5 minutes using either modality. In this paper, we qualitatively investigate the feasibility of this method and the usability of our system, and we report on some potential opportunities and challenges when using the methodology to obtain data in real-world settings.

To this end, we first conducted a one-hour usability study with 18 participants in a controlled lab setting to collect qualitative data to learn more about participants’ interactions with audio-*μ*EMA and how to build viable systems to deploy it. We used the domain of annotation of activity and posture behaviors as our example. After refining the system, we conducted a free-living study with fifteen participants, with ten of them participating for up to 21 days, to explore the feasibility of the methodology and collect quantitative and qualitative data to answer the following research questions:

- **RQ1**: What is the feasibility of deploying audio-*μ*EMA for up to seven days on a smartwatch or on a bone-conduction, over-the-ear headset?
- **RQ2**: What is the usefulness of the labels collected from audio-*μ*EMA in terms of label diversity (posture, physical activity, and context)? How consistent are these labels between different participants?
- **RQ3**: What is the audio quality of the recordings collected using *μ*EMA implemented on a smartwatch and on a bone-conduction over-the-ear headset? Can the audio-recorded labels collected *in-the-wild* be recognized by an off-the-shelf automatic speech recognition system?

The key contributions of this work are:

- We introduce the method of audio-*μ*EMA; we deployed a pilot data collection system using it, conducted a qualitative analysis of interviews, and identified four primary factors that impact the feasibility of the method: reporting in social settings, the cognitive burden of open-ended questions, the physicality of the reporting devices, and the lack of a real-time feedback system.
- We demonstrate that in a pilot study with fifteen participants, fourteen out of fifteen participants were able to sustain a response rate of over 60% for at least 7 days, and for at most 14 days.
- We demonstrate that our prototype system was able to capture diverse and temporally dense physical activity and posture labels self-reported by people in their real-world settings, with ten physical activities, eight postures, and three context label categories collected from the pilot study. Among the audio recordings collected, 95.0% of the recordings were intelligible to a human annotator.
- We discuss the implications of our findings for researchers who may want to consider using audio-*μ*EMA in future studies.

## 2 RELATED WORKS

The audio-based *μ*EMA method builds on prior work in 1) collecting in-situ behavioral data using ecological momentary assessment (EMA and *μ*EMA) and 2) using voice-based systems for EMA. We use human behavior and physical activity data collection in free-living settings as the example domain for our feasibility study.

### 2.1 Collecting *in-situ* behavioral data using Ecological Momentary Assessment (EMA)

Ecological momentary assessment, or EMA [66] (sometimes referred to as the experience sampling method, or ESM) is a data collection method used to collect information about complex, temporally dense behavioral and physiological processes in natural settings. EMA can mitigate the issue of recall bias presented in most end-of-day surveys by using notifications (that can be prescheduled or triggered using contexts) to alert the participants to report self-report data on their behaviors *in-the-moment*. EMA is a popular data collection methodology that is widely used for health monitoring research to collect data for longitudinal studies (e.g., [14, 23, 69]). The downside of EMA is that the notifications, which may be disruptive, can be perceived as burdensome; the act of pulling out a device to tap answers to questions might also interrupt the momentary behavior being measured. Balancing compensation and participants’ burden is key to ensuring good survey response rates in EMA studies. *μ*EMA is a modified version of standard EMA designed to reduce participants’ burden by restricting every survey response to a ‘microinteraction’ [33]; a microinteraction survey can be answered ‘at a glance’ in 2 to 3 s. By making it almost as easy to answer a survey as it is to ignore it, and by guaranteeing that the survey will never take more than a microinteration to answer, prior work has demonstrated that *μ*EMA can support temporally dense self-report of up to four times an hour during the waking day, sustainable for up to a year [58, 71]. Preliminary studies have suggested that it is the nature of relying on the microinteraction, not simply using a smartwatch versus a phone, that supports good compliance. Pilot work has shown that simply porting more typical multi-question survey questions to a watch from a phone is perceived as more burdensome than EMA. Prior studies using *μ*EMA to measure physical activity and other behaviors have implemented microinteraction responses using text-based, multiple-choice, single-tap-to-answer, cognitively simple questions. Limitations of *μ*EMA are the small amount of space available on the smartwatch’s screen, and the requirement for a fast, single-tap response capability – both constrain the type of self-report data that has been collected using *μ*EMA. *μ*EMA on a smartwatch, which is limited to single-tap answer options that fit on a small screen, does not provide a way to gather self-report data on all the nuanced differences between complex activities that someone might be doing throughout the day (e.g., in the domain of physical activity, “walking the dog,” “using a computer,” “scrubbing the floor,” and “playing volleyball”). One study used EMA with open-ended text-entry responses, but the interaction duration was thus relatively long (i.e., up to 5 min), and participants were only prompted three times per day [63]. *μ*EMA implemented on a smartwatch has the additional constraint that to answer a question usually requires moving both hands: turning the wrist to view the question and using the second hand to tap an answer. This can delay response time when participants’ hands are occupied, or when a piece of clothing like a long-sleeved top or jacket is covering the watch screen and must be pushed aside to see and tap the screen. *μ*EMA, like EMA, can also raise safety concerns if a *μ*EMA question is prompted in situations when it is unsafe for participants to divert their eyes to the screen (e.g., when driving or operating construction tools).

### 2.2 Audio-based EMA systems

One way to gather data on behavior passively is using passive audio sensing. Ambient sounds have been shown to provide contexts and increase performance for human activity recognition models when used as input along with accelerometer data [3, 54]. Audio-based sampling systems (e.g., [9, 19, 20, 51, 73]) have been deployed in prior studies to passively collect snippets of acoustic data from people as they go about everyday life. This method has been used to unobtrusively collect real-world data without posing any response burden. Audio recordings can include sounds from multiple environmental sources related to the targeted activities [19], which may increase the accuracy of the annotation. However, recording ambient sounds raises ethical and legal concerns [51]; the method requires safeguards to ensure the privacy of the users and people around them. Additionally, this method often requires manual annotation to make sense of the ambient noises, which can be time-consuming for large sample sizes. Audio input can also be used for self-reporting. Voice-input EMA systems prompt users who then speak to answer surveys. Audio-based systems often support open-ended input. Speech interaction can be faster than hand gestures [64], and speech input allows a participant to respond to a survey without changing activities, postures, or hand positions/movements. Well-designed voice-based interfaces may also be more accessible for older adults [60]. A recent study has used speech input from a smartwatch to collect in-situ physical activity labels from older adults [40]. Another study used an audio-based EMA system to evaluate anomia in people with aphasia in free-living conditions [32]. Voice-based EMA systems have been deployed on smartphones, smart speakers, and earables. Smartphone systems have been used to investigate speech biomarkers [43] and human activity and well-being [26]. Smart speakers have been used to measure mood and activity [77], sleep, exercise, pain level, food consumption, alcohol usage, and medication (using a recall diary) [21], and to monitor affective disorders [49]. Finally, earables have been used to capture emotional experiences in runners [16] and people with chronic back pain [17].

Researchers have explored the use of voice-based journaling in health informatics. ModEat [48, 67] is a multiplatform food journaling system that supports voice logs via conversational interaction commands. FoodScrap [46] integrates a voice-based guided journaling functionality into an *OmniTrack* [41] mobile platform to support daily food practice. TandemTrack [47] is a system combining a smart speaker with a mobile phone that supports exercise logging, reminders, and feedback using touch and speech interaction. Custom input hardware has been proposed as well; in one study a frame-shaped self-recording monitor was developed to capture everyday moods using both face-facing video recordings and verbal responses [35].

All these previous audio-input EMA/recall/journaling systems have either used a once-daily sampling rate, interactions that are too long to be considered a 2 to 4 s microinteraction (i.e., many >1 min), or were evaluated using only a short window of time during a day (i.e., 30 to 60 min), not the entire waking day. Table 1 summarizes key aspects of this prior work. The proposed methodology and system differ from this prior work in modality, input format, within-day prompting frequency, and coverage of the waking day.

The audio-based *μ*EMA system discussed in the remainder of this paper allows quick, open-ended microinteraction self-report and delivers prompts at an intensive temporal interval (as little as only 2 min between prompts). We explore the feasibility of this intensive self-report system. If such intensive responding is viable, such a system might be capable of gathering gold-standard self-report data on fast-changing, in-situ behavior such as activity and posture with hands-free input that does not disrupt ongoing activity.

### 2.3 Collecting human behavior and physical activity data *in-the-wild*

Developing algorithms that can recognize aspects of human behavior, such as physical activities, sedentary behaviors, sleep, stress, and affect, requires training data, where sensor data from devices are synchronized with labels of behaviors. Large datasets with high-quality labeling collected under the most realistic conditions are likely to lead to accurate algorithm training and the most informative algorithm evaluation. Most often, training datasets for human behavior recognition have been collected in highly controlled laboratory settings. However, models trained and evaluated using lab data may not generalize to data collected from real-world, less-controlled settings [78]; in such situations, people may have engaged in a wider range of behaviors in more natural ways that impact the nature of the signals recorded during the behaviors.

Human activity recognition (HAR) algorithm development focuses on the problem of identifying specific activities or postures from cameras or wearable sensors. Physical activity and posture recognition is an important domain for human-computer interaction and personal health informatics. Camera-based HAR typically uses single, fixed camera positions [37]. HAR using wearable, inertial measurement unit (IMU) sensors, such as accelerometers, allows for activity recognition in *any* real-world setting. However, most work on IMU-based HAR for activity and posture recognition has been conducted using relatively small lab-based datasets with limited labels, because even in research studies it is difficult to obtain temporally dense free-living self-reported labels across multiple days of activity.

There have been attempts to capture information about a person’s daily physical activities (PA) *in-the-wild* to support training better physical activity and posture activity recognition algorithms and more realistically evaluating them. One approach is to use participants’ self-report data, often acquired using an end-of-day recall survey [74, 75]. However, data collected from end-of-day recall surveys may have recall bias, which stems from the limited ability of human memory and “retroactive reconstruction” (i.e., recall can be affected by the events happening before or after the events being recalled) [69]. Another problem with recall is a lack of fidelity, especially when combined with fast-changing activities and overlapping activities [8, 44]. Significant label errors will occur at activity boundaries, and many short behaviors will be forgotten entirely. Poorly labeled data will reduce algorithm accuracy and require even more training data. To avoid recall, studies have used body-worn cameras; participants wear a camera around their neck [25, 31, 39] or their head [30] to capture a *first-person* (egocentric) narrative of their daily activities. Although this approach does not induce response burden, the ethical and privacy concerns related to continuous recording hinder recruitment. Participants may also change their daily behaviors to avoid having sensitive information captured by the camera [38], or to avoid having other people in their environment react to, be disturbed by, or be recorded by the camera [30]. Further, the smallest, most comfortable, and most discrete head- or body-worn devices only allow recording for a few hours due to battery limitations. Finally, when video data are obtained, the annotation of the video data is a time-consuming, tedious process, and the quality of the annotation data is dependent upon an annotator being able to see enough about the scene to be certain about what was happening, without any self-report information.

When evaluating the usability and feasibility of the proposed audio-*μ*EMA method, we use the domain of labeling of activity and posture for HAR as our example. The development of audio-*μ*EMA has been motivated by the challenges we and others have faced in obtaining high-quality, temporally dense free-living labeled activity and posture data.

## 3 AUDIO-BASED *μ*EMA: OVERVIEW

In this section, we describe the design decisions, the functionality, and the implementation behind an audio-*μ*EMA system. We describe the rationale for the interaction design for audio-*μ*EMA system, the prompting strategy, the choice of reporting modality for audio-*μ*EMA, and the specific implementations we chose for our study.

### 3.1 Interaction with audio-*μ*EMA

In audio-*μ*EMA, participants are prompted to report open-ended postures and physical activity via speech. The prompts are either a vibration on the wrist or a short beep in the ear. Speech input allows participants to maintain their movement and physical activity as they self-report. This allows faster and less disruptive interaction than *μ*EMA with multiple-choice questions on the watch. The short vibration and *beep* in the ear allow distinct yet discreet prompting cues to be delivered to participants. The system needs to be able to prompt participants to self-report via audio or haptic cues unobtrusively. The prompt needs to be short (~1 s) to avoid bothering participants should they choose to, or need to, ignore it, but intense enough so that participants are unlikely to miss the prompt during physical activity or in loud environments. Participants have 7 to 10 s to respond to the prompt. The recording time needs to be of sufficient duration to allow participants to provide verbose responses while minimizing the capture of unnecessary, sensitive conversational information.

During initial testing among members of the research group, we used a *beep/*vibration to mark both the start and the end of the recordings. Sometimes, participants would decide to wait for the ending cue to resume their conversation, which caused each survey to interrupt the conversation *twice*. When deploying the system in the studies, we thus removed the end-of-recording cue.

### 3.2 Different prompting modalities for audio-*μ*EMA

**Figure 1:**
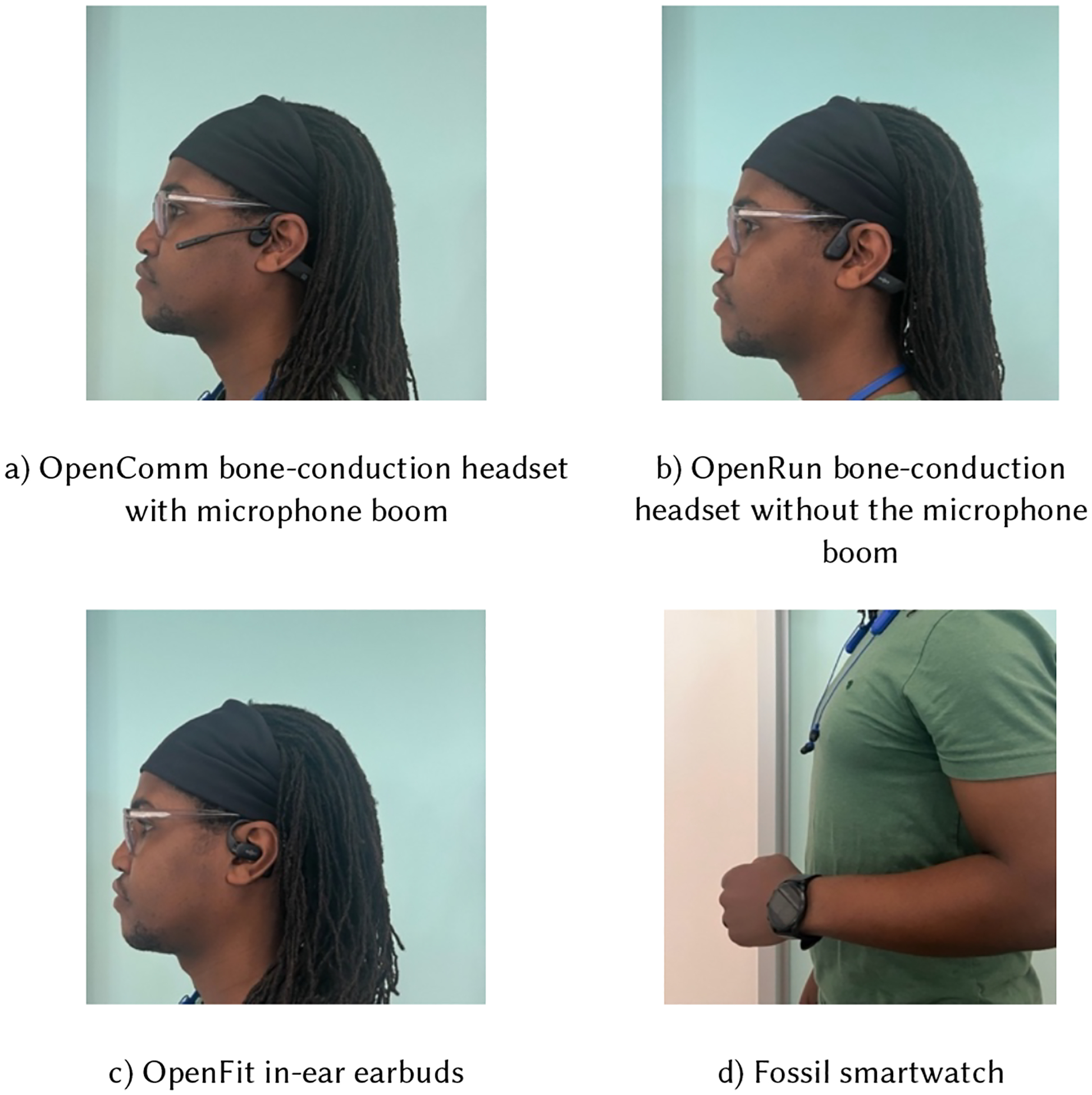
Different audio-*μ*EMA modalities.

We decided to choose earable devices and a smartwatch as recording devices for our system. Two designs for earables were considered: a bone-conduction headset and over-the-ear earbuds (Figure 1a–c). The bone-conduction headset has an open-ear design that sends sound waves through the skull instead of the eardrum and thus does not block ambient sound and is safe to use when driving, bicycling, or engaged in other activities that require listening to the ambient environment. The device also does not create pressure in the external auditory canal as some earbuds do, which some people find uncomfortable [29]. The over-the-ear design and bone-conduction technology also allows users to use their personal earbuds or headset simultaneously to listen to music and still hear the prompting. These bone-conduction devices thus have desirable properties for use in research studies that require comfortable all-day wear that permits notifying a user without obstructing normal hearing and do not prohibit the use of participants’ personal earbuds.

A smartwatch with good audio quality is also a good candidate for audio-*μ*EMA (Figure 1d). Wearing a smartwatch for a full waking day tends to be more comfortable and less noticeable to others than wearing an earable device. As our participants noted, speaking to a smartwatch is increasingly socially acceptable, so participants may draw less attention to themselves while reporting using the watch in public or during conversations. A vibration cue on the wrist, however, may be less noticeable to participants than a *beep* cue in the ear. Additionally, participants might develop a habit of bringing their hand to their mouth while reporting, which could lengthen the interaction time.

### 3.3 Prompting Strategy

Between the participants’ self-reported wake time and sleep time, the audio-*μ*EMA prompts were delivered at 2-minute to 5-minute intervals. We increased the sampling rate automatically during more intense motion (as measured by the watch) to increase the chance of capturing transitions during fast-changing activity sequences. The smartwatch estimates physical activity intensity using accelerometer data and a simple real-time algorithm that measures overall motion of the wrist. The smartwatch samples raw tri-axial accelerometer data at 50 Hz (raw signal). We smooth the raw signal using a moving average filter with a window size of 0.5 s (filtered signal). For each axis, we compute the area under the curve $AUC_{t}=\left|raw_{t}-\text{filtere}d_{t}\right|$ to compensate for the effect of gravity (DC offset for the axis). We calculate a 10 s summary of AUC by summing AUC values from the three axes to derive a coarse physical activity summary. In every 3-minute window, the PA level is considered *moderate or above* using the empirically derived threshold of 75% of the AUC values in the window being above 2,000. We determined this threshold based on pilot testing with members of the research group. When the motion is deemed moderate or greater using this heuristic algorithm, the prompting interval increases from once every five minutes to once every two minutes. We used our custom activity level estimation algorithm instead of a built-in step counter to account for moderate/vigorous activity that does not require walking around (e.g. dancing).

### 3.4 Implementation of audio-*μ*EMA

We implemented an audio-*μ*EMA system using three models of headphones. Two models used bone conduction technology, and the third used open-air earbuds. All the headphones paired with Motorola Android smartphones running Android 11+ using Bluetooth. Our goal was to test a system using affordable, consumer-grade devices that might enable cost-effective deployment in future longitudinal research studies. We used Android phones for our study because the Android operating system allows an app with appropriate user-granted permissions to gain ‘background access’ to microphones and speakers, thus supporting continuous *μ*EMA sampling. We loaned study participants a headset and a phone dedicated to use in the study; the phone had the study mobile application pre-installed through Google’s Play Store.

With the app running on the study phone and the headset paired with the phone via Bluetooth, participants received a 1 s 44 kHz *beep* through the headset; they were told this *beep* was the cue to self-report. The headsets were used without any physical or hardware modification. Audio recording started the moment the *beep* was triggered and was recorded for 7 s.

During the pilot study with members of the research team, we presented the participants with three different models of Shokz (Shokz, Inc.) [79] headsets: OpenRun (bone-conduction headset with the microphone embedded in the casing), OpenComm (bone-conduction headset with a boom microphone that could be flipped down near the mouth), and OpenFit (open-ear wireless earbuds that wrap around the ear for stability) (See Fig. 1). The devices weigh 29 g, 26 g, and 8.3 g (per bud), respectively. We advised participants to wear both buds for the OpenFit.

We implemented an audio-*μ*EMA smartwatch application using a Fossil Gen 5/6 smartwatch running Android 9 and above. The smartwatch application was paired with the same mobile application on the phone described above, but because we could program a watch application to control the prompting and recording directly, participants did not have to carry the study phone with them throughout the day.

We conducted a controlled lab measurement to explore the best-case audio quality we might expect to obtain from the three different audio-*μ*EMA modalities (i.e., Fossil smartwatch, OpenComm headset, OpenRun headset) under different levels of background noise and wear conditions (see Appendix A). Results from the experiments show that the headsets worn properly on the ears appeared to have better audio quality than the smartwatch. However, the difference between the audio intelligibility of the headset with the microphone boom (OpenComm) and without (OpenRun) across different wear conditions was modest.

During the internal testing round, and the usability study, we noticed that the wireless earbuds had significantly poorer battery life and more connection issues than the bone-conduction headset. After considering the trade-offs between different modalities, *we decided to deploy the bone-conduction headsets and the smartwatch in the feasibility field study*.

## 4 STUDY DESIGN

We conducted two pilot studies to explore the feasibility of audio-*μ*EMA design options. The first study was a one-hour usability session during which each participant (1) was trained on using the technology for 10 min, (2) used the system in a semi-naturalistic setting, and (3) provided impressions in a semi-structured interview. The second study was a free-living, within-subject feasibility study during which each participant (1) was trained on using the technology for 10 min, (2) used the headset-based system for 2 to 7 days, (3) used the smartwatch-based system for 2 to 7 days, (4) used standard multiple-choice *μ*EMA on a smartwatch for 2 to 7 days, and then (5) provided impressions in a debriefing session. Thirteen out of fifteen interviews were conducted in person; the other two were conducted over Zoom. All in-person interviews were recorded using a hand-held recording device, and the remote interviews were recorded using Zoom.

### 4.1 Participant recruitment and procedures

Our study protocol was approved by the institutional review board at Northeastern University. We recruited participants via emails, social media posts, and flyers posted around the university campus. Individuals were eligible to participate if they (1) were between 18 and 55 years of age, (2) had no cognitive or hearing impairments, (3) were willing to wear a smartwatch, wear headset, and carry a second phone with them from between one hour to 28 days, and (4) did not need reading glasses to read their phones. Participants could participate in either one or both studies; four participated in both. All participants provided informed consent. Participants in the usability study were compensated with $20, and participants in the free-living study were compensated with $20 for the initial interview, $20 for the exit interview, and $5 for each day they wore the devices. Compensation was delivered using online gift cards. Participants were told the study’s purpose was to collect motion data and activity and posture labels to train better activity recognition models.

### 4.2 Usability study

After consenting to the study, participants were invited to an in-person, one-hour usability session. We collected demographic data, including age, occupation, self-reported technical proficiency, and daily physical activity levels. A research assistant introduced the AudioEMA mobile application and demonstrated how the different headsets are worn. Participants were asked to try on different headset models, provide their initial impression of the headsets, and choose which model they would like to wear during the one-hour session. We mentioned to the participants that the mobile application is used to send signals to the headsets and transfer audio recordings to a secure server. The research assistant guided the participants on how to put the headset on, how to recognize the reporting cue (the 44 kHz 1 s “*beep*”), and how to report their posture and activity when the *beep* is heard. We provided the participants with a list of postures and physical activity examples (see Appendix B). We instructed the participants that their response should be the posture and physical activity they were engaged in *at the time of the beep*, e.g., if the participant was in a dance class, but the *beep* came while standing still, the desired response would be “*standing in a dance class*” and not “*dancing*.” Participants could describe their posture and physical activities in different ways; what was important was that they included the posture and the physical activity in their response. This training typically took ~10 min. After training, we asked the participants to wear the headset, carry the phone, and answer the cues throughout the remainder of the one-hour session, even during the semi-structured interview.

After any clarifying questions, the participants performed three ~10 min simulated scenarios that caused them to engage in different postures and activities (see Table 2). While going through the scenarios, participants wore the headset, carried the phone, and had 7 s to report their activity and posture every time they heard a *μ*EMA prompt, once every 3 min. Finally, the participants filled out the System Usability Survey (SUS) [18] and answered some questions in a 20 to 30 minute interview.

### 4.3 Feasibility study: Within-subject free-living study

After collecting qualitative feedback from the usability study and making appropriate changes to the systems and the study protocol (see Section 5), we conducted a longer-duration, free-living field study to better assess factors that might impact the feasibility, compliance, data quality, and sustainability of different versions of the system when used in real-world conditions. Participants were asked to test three different data collection systems for an equal duration of days, each for the entire waking period of each day: (1) answering *μ*EMA prompts via speech using either the boom or non-boom bone-conduction headset (randomly-assigned) every 2 to 5 min, (2) answering *μ*EMA prompts via speech using the smartwatch every 2 to 5 min, and (3) answering multiple-choice *μ*EMA prompts using a tap action on the smartwatch every 15 min (we implemented this approach based on prior work as a baseline [33]). Participants had to carry the smartphone with them in a pocket or bag or keep it nearby when at home, wear the smartwatch during their waking day, and charge the phone and headset each evening. For one third of the study, participants had to wear the headset during their waking day. We asked the participants to provide a self-report response whenever they heard the *beep* from the headset or a beep/vibration from the smartwatch. Given this is exploratory work and the tolerance for such a high sampling rate via an audio system was unknown, we started by conducting shorter duration studies (two days per condition, six days overall) and gradually increased the study duration (first, four days per condition, 12 days overall and then seven days per condition, 21 days overall).

#### 4.3.1 One-hour introductory session

After obtaining informed consent from participants for the free-living study, we invited participants to an in-person, one-hour introductory session. If the participants were not in the first usability study, we collected demographic information from the participants. A research assistant introduced the AudioEMA mobile and wear application, and a headset, to each participant. Unlike the usability study, participants were assigned a headset model (OpenComm or OpenRun). We provided the participants with a Motorola smartphone, a Fossil smartwatch (Gen 5/6), the assigned headset, and device charging cables. The research assistant guided the participants through using the devices, recognizing the reporting cues, and answering the prompts. During the training session, we defined physical activity as “*what you are doing the moment when the beep goes off*.” However, during the six-day version of the free-living study, we noticed that participants tended to report ambiguous physical activity labels. For example, many participants would report “*sitting, working*” without providing the context for the task they were doing (e.g., “*working*” could mean “*using the computer*,” “*doing paperwork*,” or “*in a meeting*”). Ambiguous labels can decrease the accuracy of an activity recognition algorithm because “*using the computer*” and “*in a meeting*” can generate different sensor signals, even though they both fall under the category of “*working*.” In the longer duration iterations of the study (12 and 21 days), we asked participants to provide context to their labels, if possible. For example, instead of simply reporting “*typing*,” we encouraged them to indicate which device they were typing on, like “*typing on laptop*” or “*typing on phone*.”

#### 4.3.2 Field study protocol

**Figure 2:**
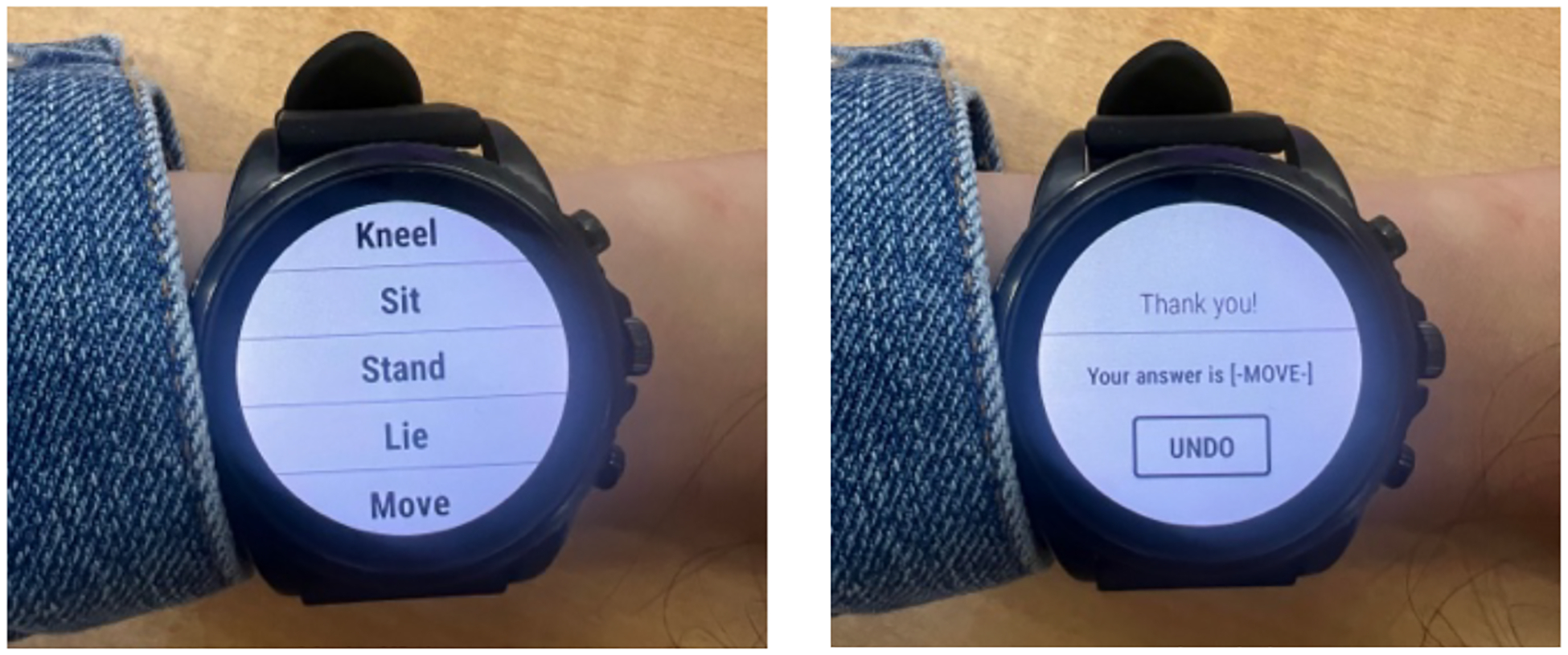
Multiple-choice *μ*EMA question on the smartwatch.

During the field study, participants used the three different data collection systems for an equal amount of time (2 to 7 days per condition): (1) a Shokz headset that delivered an audio-*μ*EMA prompt (a *beep* in the ear) once every 2 to 5 min and allowed speech input, (2) a smartwatch that delivered a *μ*EMA prompt (a *vibration* on the wrist) every 2 to 5 minutes and allowed speech input, and (3) a smartwatch that delivered a *μ*EMA prompt (a *vibration* on the wrist) every 15 min and allowed tap input to answer a single multiple-choice question. The three study conditions were randomized for the 21-day deployment of the study. We present the multiple-choice *μ*EMA question prompted on the smartwatch in Figure 2; this approach is consistent with prior work by Ponnada et al. [58]. The devices prompted the participants between their self-reported wake and sleep times. We also instructed the participants to wear the devices while they were awake (waking day), and to only take off the devices when they were asleep, in the shower, swimming, or in dangerous situations like driving. Participants could ignore the prompts in certain stressful situations (e.g. job interviews, vigorous physical activity, workplace emergencies, intimate/heated conversations). We also asked the participants to connect the phone to their home Wi-Fi network (to facilitate data transfer to the study server), answer a daily survey on the phone, charge the devices, and keep the watch and the phone connected every night during sleep. A research assistant sent a daily text message to the participants reminding them of which device they were using that day and the tasks they needed to do (i.e., charging). Participants in the free-living study commented that real-time feedback from the phone and on-demand help from the researchers helped overcome this technical issue: “*There was a day when it (the headset) wasn’t connected, and I struggled to figure that out. I think I was able to contact you … and it was resolved pretty quickly. I think having a researcher that you can contact and be like, ‘Hey, this is not working for me right now’ was really helpful*.” [F8]

The *μ*EMA multiple-choice questions on the smartwatch were prompted at 15-minute intervals. We chose the same prompting interval proposed in prior work [59]. Each *μ*EMA question appeared for up to 15 s, or until answered, on the smartwatch.

#### 4.3.3 Exit interview

After concluding the field study, we invited the participants for an in-person, one-hour interview. The interview had two parts: a previous day recall adapted from the 24PAR physical activity recall method [42], where the participant would recall their postures and physical activity from the morning of the previous day to the beginning of the interview; and a semi-structured interview where participants provided their overall impressions of the reporting systems.

#### 4.3.4 Ethical concerns and considerations

As part of obtaining consent, we informed the participants that they would be recorded for up to 10 s per audio-*μ*EMA prompts (using the headset or watch) and that background/sensitive conversations could be accidentally recorded in the audio clips. We indicated that only the trained research team would have access to the audio clips, and they would only be used for this study.

To limit bias and confounding results, the consent process and consent form did not indicate that the true purpose of the study was to measure compliance, perceived burden, validity, and survey responsiveness. Instead, we told the participants that they were participating in a study to collect activity and posture labels and sensing data from wearable devices to develop new activity recognition algorithms. During the debriefing process at the end of the study, we notified the participants of the true purpose of the study.

### 4.4 Participants overview

Across both studies, we had 29 participants test the prototype systems: 14 participants took part in the usability study, 11 participants took part in the feasibility free-living field study, and four participants took part in both studies. One participant from the free-living study withdrew after two days in the study due to illness and was removed from the data analysis. We present the participants’ demographic information in Table 3. To avoid confusion between results from two studies, in later sections of the paper, we use the prefix U with a number to denote participants from the usability study, and the prefix F with a number to denote participants from the free-living study.

## 5 RESULTS

In this section, we report quantitative and qualitative results from the usability and feasibility study. In Section 5.1, we investigate the feasibility of deploying audio-*μ*EMA in free-living settings (**RQ1**) by comparing the response rate and perceived burden of participants between audio-*μ*EMA on the smartwatch and headset vs. *μ*EMA on the smartwatch (baseline system). In Section 5.2, we categorize the list of labels collected from audio-*μ*EMA and show the diversity and the consistency of different posture and activity labels collected using audio-*μ*EMA, which demonstrates the potential of collecting realistic labels using this data collection method *in-the-wild* (**RQ2**). In Section 5.3, we investigate the audio quality of recordings collected using the watch and the headset (**RQ3**). We discuss the list of background noises recorded in the audio clips and how they affect the audibility of the recordings and present the accuracy of the transcription generated by an off-the-self speech-to-text model. Section 5.4 summarizes the qualitative feedback we received from participants during the exit interview of the usability and free-living studies.

### 5.1 Assessing the feasibility of deploying audio-*μ*EMA *in-the-wild* (RQ1)

We investigated the feasibility of deploying audio-*μ*EMA for up to 7 days on a smartwatch or a bone-conduction headset measuring compliance and perceived burden.

#### 5.1.1 Prompt-response rate

In the free-living feasibility study, three participants used the prototypes for six days (two days per condition), two participants for 12 days (four days per condition), and five participants for 21 days (seven days per condition). We describe the rate at which participants answered prompts using two metrics: completion and compliance rates. Completion is defined as:

$$\text{Completion}\%=\frac{\text{PromptsAnswered}}{\text{PromptsDelivered}}\times 100$$

Compliance is defined as:

$$\text{Compliance}\%=\frac{\text{PromptsAnswered}}{\text{PromptsScheduled}}\times 100$$

*PromptsAnswered* in the audio-*μ*EMA conditions refer to the audio recordings where, based on human review of the audio, the participants attempted to respond to the prompts; a response could meet this standard even if the full response was not intelligible, such as when an annotator could make sense of the posture but not the physical activity. Audio recordings that were overpowered by background noises or where the participant was not heard speaking and attempting to answer were not counted as answered prompts. If we heard the participants’ voices in the recordings but no posture or activity was reported (e.g., participants were talking to someone), the prompt was not counted as answered. Two researchers independently coded the responses for 1,200 audio clips. The inter-rater reliability rate for *PromptsAnswered* rate is *k*=0.99, suggesting a high rate of agreement between annotators [34].

*PromptsDelivered* in the audio-*μ*EMA conditions refer to the total number of audio recordings collected. *PromptsScheduled* per day in the audio-*μ*EMA conditions were estimated by measuring the number of hours between the participant’s self-reported wake and sleep time, multiplied by 12 because 12 was the minimum number of audio-*μ*EMA prompts scheduled per hour (i.e., one every 5 min).

*PromptsAnswered* in the *μ*EMA condition refers to prompts where a participant clicked on one of the multiple-choice responses. *PromptsDelivered* in the *μ*EMA conditions refer to the total number of prompts that successfully appeared on the participant’s watch display. *PromptsScheduled* per day in the *μ*EMA conditions were calculated by measuring the number of hours between the participant’s self-reported wake and sleep time, multiplied by four because four *μ*EMA prompts were scheduled per hour).

Table 4 shows the completion rate for each participant across the three conditions. On average, there were 87.7 answered audio prompts from the headset collected per day (SD = 31.2, range = 25–148); 95.2 answered audio prompts from the watch per day (SD = 36.7, range = 27–188); and 29.4 answered multiple-choice prompts from the smartwatch (SD = 10.3, range = 4–54). Due to a technical issue with the software, we lost one day of headset recording for F6 and F8, and two days of *μ*EMA responses from F15. We lost three days of watch audio data from F6 and one day of watch audio data from F14 because the participants forgot to bring their watch chargers during travel.

Overall, days with *μ*EMA condition had a higher completion rate than days with audio-*μ*EMA on the headset (t=2.38, p<0.001) and audio-*μ*EMA on the smartwatch (t=3.8, p<0.001). Audio-*μ*EMA on the watch had a slightly higher completion rate than audio-*μ*EMA on the headset, but this difference is not statistically significant. Despite the intensive prompting frequency, 13 out of 15 participants were engaging with the audio-*μ*EMA system on the headset with a >60% completion rate; of the remaining two, F6 reported having technical difficulty keeping the headset connected to the phone, and F1 had a client-facing job and was not allowed to wear the headset while interacting with customers. Thirteen out of 15 participants were also engaged with the audio-*μ*EMA on the smartwatch with >60% overall completion rates, except for F1 and F7. F7 reported in the exit interview that they had trouble remembering to charge the watch at night and put it on in the morning, which decreased the completion rate in the watch audio-*μ*EMA and *μ*EMA condition.

The low compliance rate in the *μ*EMA condition is due to the poor battery level on the watch (the watch stopped prompting *μ*EMA if the battery was lower than 20% capacity), causing a large amount of *μ*EMA prompts (18.5%) to fail to be delivered.

We used a mixed-effect linear model to investigate the differences in the trend in completion rate between the three conditions. We set the significant level to 0.05. The equation shows the template for our mixed-effect model:

$$\text{CompletionRate}\sim \text{StudyCondition}*\text{DayIntoStudy}+\left(1|\text{Participant}\right)$$

**Figure 3:**
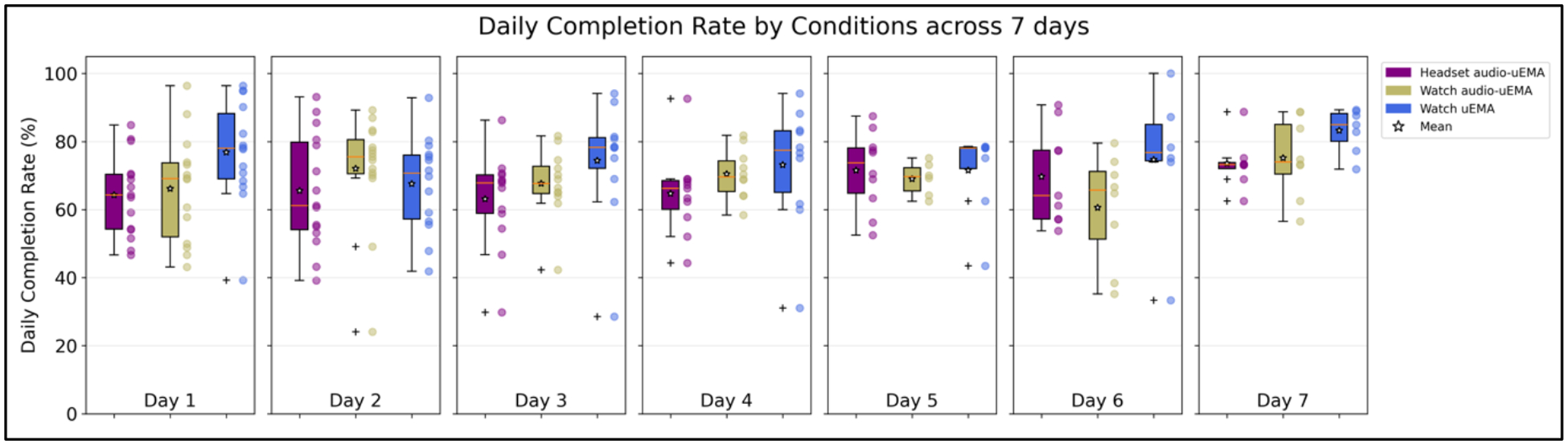
Trend in daily completion rate by day into study across three conditions.

Overall, the response trend stays above 50% across 7 days for all three conditions. Figure 3 shows the completion trend across 7 days of the study between three study conditions. The completion rate increases across 7 days for all three conditions, which we attribute to the learning effects of the first one or two days drawing the response rate down. Although the slope for the headset audio-*μ*EMA and the *μ*EMA on the watch conditions were steeper compared to the audio-*μ*EMA on the watch, we did not find any statistical differences in the response rate trend between the three groups.

#### 5.1.2 Daily perceived burden

Participants answered a daily burden survey on the phone. The questions used to measure burden included: (1) “The smartwatch/headset is easy to learn how to use,” (2) “I learned to respond to smartwatch/headset prompts quickly,” (3) “I easily remember how to respond to smartwatch/headset prompts,” and (4) “I feel comfortable wearing the smartwatch/headset.” All questions had five answer choices, ranging from “Strongly Disagree” to “Strongly Agree.” The survey also asked whether the smartwatch/headset needed to be recharged at any point during the day with answer options “Yes” and “No.”

**Figure 4:**
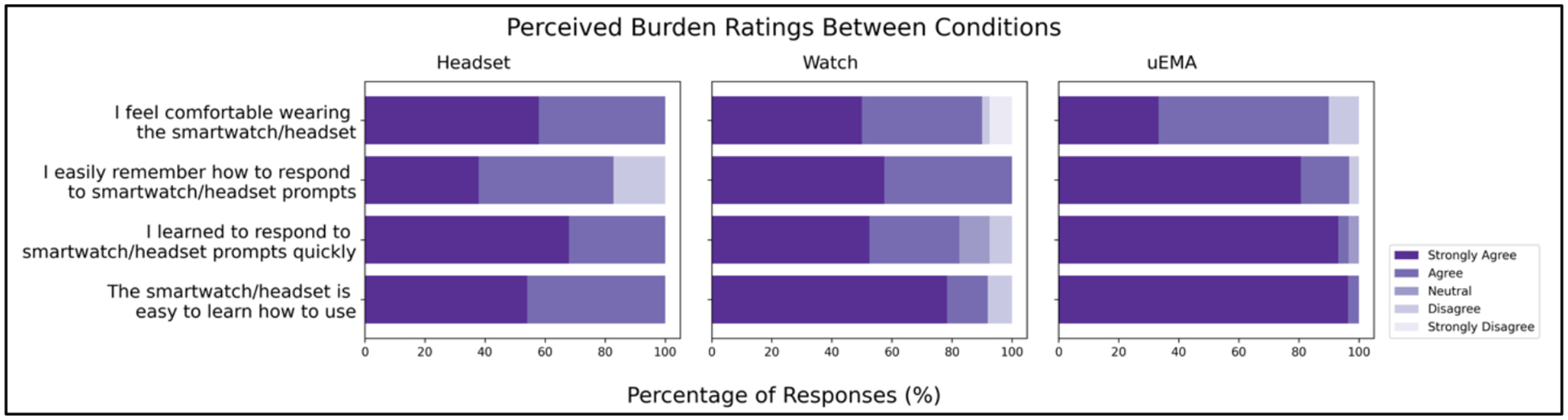
Results from the daily perceived burden survey.

We received 114 end-of-day responses from participants (response rate of 52.1%; 114/219). Figure 4 shows the results of the perceived burden survey responses across the three conditions. Overall, participants “agreed” or “strongly agreed” that wearing the devices felt comfortable. Few “disagreed” that remembering how to respond to prompts was easy on the headset audio-*μ*EMA (17.9%; 5/28 responses) and the watch-*μ*EMA (5.6%; 2/36 responses) conditions. Only one participant “disagreed” that the watch is easy to learn how to use (2.3%; 1/44 responses).

We conducted Kruskal-Wallis tests to check for significant differences between the three conditions for each of the perceived burden questions. We found that there is a significant difference for the “easy to answer” question (*χ*^2^ = 161.9, p<0.001). We conducted post-hoc analysis using the Wilcoxon rank sum test with Benjamini & Hochberg adjustment [15] and found that participants reported answering *μ*EMA questions was easier than both audio-*μ*EMA on the watch and the headset. Similarly, we found a significant difference for the “easy to learn how to use” question, with participants reporting they felt that that the condition *μ*EMA and audio- *μ*EMA on the watch was easier than audio- *μ*EMA on the headset (*χ*^2^ =148.9, p<0.001). We also found significant differences in the “respond to prompts quickly” question, with participants reporting they felt that condition *μ*EMA was responded to more quickly than both audio- *μ*EMA on the headset and watch (*χ*^2^ =156.4, p<0.001). We did not find any significant difference between the three groups for the “comfortable to wear” question (p=0.72).

Ten out of 45 responses (22.2%) indicated the headset needed to be recharged in the middle of the day, but 48 out of 69 responses (69.6%) indicated that the watch needed to be recharged during the day.

#### 5.1.3 System usability scale (SUS)

We collected 15 responses to the System Usability Scale survey during the usability study. We did not add the SUS into the usability session until participant F4 (the first three participants did not do the SUS survey). We converted the Likert responses to the items in the survey to numeric values from 0 to 4 and multiplied the total values by 2.5 [18]. The obtained mean score was 81.67 with a standard deviation of 7.95. This suggests a high level of perceived usability among the participants and a favorable user experience [10].

### 5.2 Investigating the consistency and diversity of labels collected using audio-*μ*EMA (RQ2)

Two researchers collectively listened to the 21,595 audio clips recorded in the usability and feasibility studies; they each transcribed the labels identifiable from the audio recordings. They independently recorded whether the participant answered the prompt and whether the response was intelligible to a human annotator.

After this transcription, a researcher grouped 937 unique labels into seven postures, ten activity categories, and three context types. Table 7 shows the list and summary of the postures, activities, and context types extracted from the audio recordings.

Our system was able to record a wide variety of different postures, activities, and contextual labels. Using speech input and micro-interaction, our system was able to keep participants engaged even with intense prompting intervals. Among the participants in the free-living study, only one participant (F2) consistently reported only the posture (with no additional context or physical activity). During the exit interview, the participant reported that he forgot that he had to report the activity and the posture. We modified the instruction during the first meeting with participants to emphasize that the research team is looking for two components in the verbal response: a posture and an activity (additional contextual information is encouraged but optional). No subsequent participants reported the same issue as F2.

However, due to the open-ended input format of the input labels, different participants displayed different reporting behaviors, which is shown through the various variations of labels in Table 5. For example, “*sitting*” could have multiple variations, such as: “*sit*,” “*sit upright*,” “*sitting upright*,” “*sitting down*,” and “*sit on the floor*.” Participants also interpreted the same labels differently, based on their daily routines. For example, F5 used “*moving*” to indicate postures during dancing practice, while F4 used “*moving*” to indicate postures during body weight exercises. The inconsistency in reporting patterns between participants could prove difficult if we want to use the labels to train activity recognition models.

### 5.3 Investigating the audio quality of the recordings collected using audio-*μ*EMA (RQ3)

Two annotators independently annotated the audio quality of the audio recordings in two ways: (1) the types of background noises heard in the audio clips, and (2) the intelligibility of the recordings to the human annotator. Both annotators coded the same 1,200 audio clips, and the inter-rater reliability rate (Cohen-kappa) for background noise is k=0.86 and the intelligibility is k=0.99, which indicates substantial agreement between annotators. Table 6 lists the background noise categories we identified from all audio recordings. Overall, 69.2% (14,933/21,595) of all audio recordings contained background noise (45.7% of the headset recordings, and 51.2% of the watch recordings). The most common type of background noise captured in the audio clips was “*conversation*” (48.3%), which can indicate noises from conversations that the participants were actively a part of, or background conversations in public places.

Overall, 3.8% (381/10,608) of headset recordings and 1.8% (105/10,987) of the watch recordings were deemed unintelligible. During the annotation process, we noticed that audio clips with loud weather-related noises (strong wind or heavy rain) or music (e.g., when participants were attending concerts or at bars/clubs) were the most difficult to transcribe, and participants had to raise their voices for the recordings to be intelligible. Audio clips with “*conversation*” as one of the background noises tend to have a higher non-response rate compared to other audio clips, which may indicate that participants are less likely to respond to audio-*μ*EMA prompts in social settings.

To investigate the possibility of automatically extracting activity and posture labels in real time, we measured the accuracy of Google’s speech recognition API^1^ using the human annotator’s label extraction results as the ground truth. We chose this speech recognition API because it can be run locally on Android devices in real time. We computed the accuracy of the speech recognition models programmatically. The human annotators’ labels and the speech recognition’s output were stemmed and lemmatized (e.g. “*sitting*” was converted into “*sit*”). We programmatically determined the accuracy by checking if the human annotator extracted physical activity and posture labels as a substring of the speech recognition’s output. For example, if the human annotator’s extracted posture/physical activity label was “*sitting*”/”*on my phone*,” and the speech recognition result was “*sit and on my phone*,” results from speech recognition were marked as accurate for both posture and activity/context. Overall, the accuracy of Google speech-to-text on all intelligible audio recordings was 22.5% for posture and 23.3% for physical activity. Table 7 shows the details of Google’s speech recognition performance on watch and headset intelligible recordings, broken down by the existence of background.

### 5.4 Participants’ experiences with audio-*μ*EMA

In this section, we present the qualitative results from the usability and feasibility studies. We have identified four primary factors that hindered the compliance/completion rate when deploying audio-*μ*EMA in real-world settings.

#### 5.4.1 Verbal reporting around other people

When asked about when verbal reporting was inconvenient, thirteen out of fifteen participants in the free-living study and 16 out of 18 participants in the usability study expressed concerns about doing the self-reporting when around other people. Five out of seven participants in the feasibility study said answering the prompts during a conversation was minorly disruptive because it could draw focus away from the conversation. However, the extra time needed to explain the purpose of their reporting to another person nearby can increase the duration of an interaction. Five participants mentioned that even after explaining the study once, people around them still paid attention to their subsequent report attempts. However, no participants mentioned that people around them were annoyed by the prompting. Talking about this, F7 said:

… I would be talking to my friends, and I’d get the prompt. And I’d answer the prompt and they were like ‘*What are you doing?*’ and I’d be like ‘*I told you that I was doing this for a study, like leave me alone*’. Some people find it funny, like my coworkers at work. I’d answer the prompts and they’d be like ‘*Are you that bored that you’re just stating what you are doing?*’ and I’d be like ‘*I told you I did this for a study*.’

In some situations that require silence and/or attention, participants indicated that they did not answer prompts. Scenarios listed under this category include: “*important meetings*” [F5], “*wedding ceremony*” [F7], “*during a presentation*” [F3], “*on a plane*” [F8], “*talking to clients*” [F1,8], and “*in class*” [F6]. Answering the prompts during these situations can draw unnecessary attention towards the users and be seen as “*rude*” or “*not paying attention*.” Talking about this, F3 said:

I was doing my hackathon and one day we had to present, and people were talking about their project, and they were doing the presentation. There are not many people in the hall, like the judges and the teams. So, it was very quiet. And if I say something, that would draw attention [paused] and like, [be] considered rude that I’m talking when someone is presenting.

Participants did not report having similar issues with multiple-choice *μ*EMA, since tap interaction on the watch was seen as more “*socially acceptable”* (F8). However, ten out of 15 participants in the field study agreed that they preferred to do the verbal report when they were alone or during vigorous physical activity. Talking about this, F13 said: “*I think saying it verbally can be more convenient when like you’re on the bus and you don’t have a lot of room to move your hand, or if you just want to do it quick and not later, like go for a run or lifting weights.”*

#### 5.4.2 Cognitive burden from verbal, open-ended responses

Initially, during the usability session, three participants expressed their concerns about the cognitive burden of verbal reporting during focused time (e.g., doing homework, coding, doing paperwork): “*I’ll say I’ll still be annoyed during the homework because you know, like doing assignments, and that’s basically you need to concentrate on that stuff*” [U6]. However, during the free-living study, fourteen out of fifteen participants reported no discomfort doing verbal reporting during focus time: “*I don’t think the headset and the watch itself are that disruptive to my focus [paused] like it becomes second nature – I just say it and continue whatever I was doing*” [F5]. When comparing open-ended audio-*μ*EMA with multiple-choice *μ*EMA on the smartwatch, eight out of fifteen participants expressed that there was a small cognitive burden in coming up with the response: “*It takes a little bit more brain power, I think, it’s not like it’s a lot, but you have to be self-conscious of what you are doing and then speaking into it [the watch] versus … like it can be three in the morning and I can just tap ‘LIE’ and I don’t have to think twice about it*” [F1]. The burden can also increase when a prompt is delivered during an activity transition period: “*I would be transitioning from one position to the next, and then I would get a prompt like right in the middle of me transitioning and I wouldn’t know which one to go with*.” [F7]

During the one-hour usability session, eleven participants expressed their annoyance over having to repeat the same response when they were doing similar activities for an extended period: *“… because if I do something I don’t switch between what I do. So overall I just keep on answering the question and giving the same answers*.” [U4]. We thus asked if they would want a keyword (e.g., “same” or “same thing”) that could be used to indicate that they are doing the same activity and posture as in the previous prompt; fourteen participants reacted positively to this option. U14 speculated that the keyword would reduce the cognitive burden in producing the response: “*… if I was just like sitting and writing for a while, then if I keep saying ‘sitting writing’ I might get annoyed … saying the word ‘same thing’ is easier in terms of like thinking wise*.”

In response to this feedback in the usability study, during the free-living study, we told participants they could use the “same” keyword to simplify their reporting. However, we then noticed that participants were overusing the “*same*” option and might have forgotten to report activity/posture transitions (on one day, F4 repeated the *“same”* label for 637 min consecutively). We also found that we lost data when the recording of the activity is unintelligible due to background noise and then it is followed by “*same*” reports (there were 86 cases out of 2,416 (3.6%) with inaudible/non-response recordings before “*same*” labels). We therefore removed the “*same*” option for F8–15 and do not recommend its use.

#### 5.4.3 Participants’ concerns over audio quality

Six out of fifteen participants in the free-living study expressed their concerns over the audio quality of the verbal responses that they were providing to the research team, especially on the watch. All six participants stated that because they feared their responses might not be recorded, they tended to bring the watch close to their mouth when they answered the prompts: “*I was a bit skeptical if my voice is reaching to the watch or not, then I have to take the watch closer to my mouth every time*” [F2].

Participants further expressed they sometimes choose to ignore the prompts or take off the devices completely if they felt their answers might not be audible to the researchers in loud environments: “*I was out with my friends in a club or a concert and I would get the vibration … I would try to bring it close to my mouth so that hopefully you can hear me, but I wasn’t sure if that worked. Then after that I was like I don’t think they are getting anything from me, so I didn’t really see the point*” [F7]. Five participants mentioned that they wanted to be able to play back some of the audio to make sure that their voice was audible to the researchers: “*For me I need to hear it once … like what one audio clip sounds like and then I would feel more comfortable*” [F5].

#### 5.4.4 How the reporting modality affects participant’s comfort level and adherence rate

During the usability session, no participants mentioned specific concerns over the comfort of the bone-conduction headset without the boom (OpenRun): “[The OpenRun] *sticks around, and this is just like I’m wearing nothing … I don’t have something inside my ear. You just wear it*.” [U1]. However, eleven out of fifteen participants in the free-living study mentioned that the design is painful to wear for an extended period and during certain positions like “lying down” or “reclining on a chair.” Some participants mentioned that they tended to switch between different wear conditions to avoid discomfort – including wearing the device around the neck or on one ear (i.e., in ways the research team never anticipated): “*The headset … after wearing it for a long time, my ears were kind of irritable … But when I put it around my neck, it was fine*” [F7]. This change in wear style can impact the audio quality of the recordings, however, and the headsets do not provide a way to programmatically know or record how they are being worn.

Eleven participants in the usability study and twelve out of fifteen participants in the free-living study expressed strong preferences for the smartwatch as a reporting modality. Seven participants in the usability study and four participants in the free-living study commented that wearing the watch is more comfortable than – and less noticeable than – the headsets: “*The watch is a lot better, even speaking to the watch was a lot better than the headset just because it was more comfortable to wear, it was more natural, like you could wear it around in public and around my friends and you wouldn’t be like ‘What are you wearing?’”* [F9]. Five participants in the usability study and three participants in the free-living study also commented that verbal interactions with the watch are becoming more socially acceptable: speaking to the watch would not draw attention in public settings while wearing a headset could make them look rude during a conversation. Talking about this, U18 said:

I feel like it’s more professional too, because like when I’m working, I guess people may think like oh, she’s listening to music or something like oh, she doesn’t have her full attention to me […] But for the watch, I think it’s a very common thing for people to like look at their phone, like briefly or like, look at their Apple Watch briefly. So I feel like it would be more natural to do in a social environment.

In the exit interview for the free-living study, we asked participants whether they would have been willing to do a context-assistive recall diary [61, 65] by the end of the day to fill in non-response prompts, either on their phone or on a computer program. Four participants said they were willing to do the recall. F7 suggested that instead of doing the recall at the end of the day, she wanted to be able to do the recall throughout the day: “*I think it would be better if … I just fill it out like, I don’t know, like an hour after, or like 30 minutes later. I don’t know if I could do everything at night. I think as I go through the day, whenever I get the chance, I think having that option is better*.” [F7].

## 6 DISCUSSION

In this section, we discuss the implications of our results on the viability of the audio-*μ*EMA method.

### 6.1 Feasibility and usability of audio-*μ*EMA

Results from the usability and the feasibility study showed that despite the high prompting frequency of once every five minutes or more (12 times per hour), participants were able to respond to audio-*μ*EMA prompts up to 14 consecutive days during their self-reported waking hours, with a relatively high completion rate of 67.7% and compliance rate of 60.9%. The overall completion rate of *μ*EMA on the smartwatch was higher, at 73.9%, but at one-third of the sampling rate (4 times per hour). This demonstrates the feasibility of using audio-*μ*EMA to collect temporally dense open-ended labels in free-living settings **(RQ1)**. Our preliminary testing within our research group with *μ*EMA on the smartwatch at rates of 12 times per hour felt unsustainable, so we did not test the feasibility of that rate; testing with audio-*μ*EMA did feel manageable at 12 times per hour, which led to this usability and feasibility pilot study. Overall, aside from unusual situations such as traveling, participating in a wedding ceremony, or taking an exam, we found that our participants were willing to wear the devices and answer prompts, even in public settings and around other people.

There were device hardware limitations that negatively impacted the response rate, such as limited battery life and Bluetooth connectivity. The physicality of the devices could also cause some discomfort, negatively impacting the response rate. Perhaps the most significant challenge with the audio-*μ*EMA reported, however, was the social burden [46, 47] for participants in client-facing roles such as clinicians or people working in hospitality. For some participants, even just wearing head-mounted or earable devices, let alone speaking aloud regularly, was forbidden at work or when interacting with clients. A second significant challenge participants reported resulted from the open-ended nature of the verbal response, which may induce cognitive burden [46] on the participants, increase response time, and dampen the microinteraction quality that makes the high temporal density reporting sustainable. Finally, the lack of real-time feedback when using an auditory input mechanism with no display created some concerns among participants, sometimes in ways that negatively impacted usability and data availability.

Device form factors can also be a factor that influences adherence and the quality of the actual accelerometer signal recorded across different activities. Participants reported that the headset design makes it difficult to wear during lying or reclining positions because the back of the headset irritated the back of participants’ heads. Certain activities also require the removal of the headset, such as “*changing clothes*” (F13), or “*putting on [a] VR headset*” (F9). Although the watch can be worn comfortably throughout a waking day, participants also reported that they tended to bring the watch closer to their mouths when reporting, which potentially affected the quality of the accelerometer data signal being recorded by the device. If participants default to bringing the watch to the mount, it may reduce the response rate when doing activities that use the hands, such as carrying groceries or weightlifting. Two participants commented that they took off the watch in quiet environments because the sound of the vibration was audible to people around them.

Nevertheless, despite the challenges, the experience of using the system was generally tolerable, with participants using it for up to 14 days with a response rate of 67.7% across all participants, and the quality of the label data obtained appears to be sufficiently high to warrant further study.

### 6.2 Usability Concerns with Consumer Devices

From the qualitative findings of our study, designs of prompting/recording devices have an impact on the participant’s comfort level and might have an impact on the adherence rate of the participants. However, with the headset models we used in our study, there is no *ideal* option in terms of both comfort level and research quality.

To be usable in a research study, an *ideal* prompting device should have good audio quality to capture users’ verbal reports, even with loud background noise and low speech volume. The battery level of the device should last for a full waking day (16+ hours) to avoid data loss. The device should be able to *automatically* reconnect with the phone if it goes out of Bluetooth range; if automatic reconnection is not possible, it should send an audio signal to the participant to manually reconnect to the phone. Ideally, the earable device would be capable of recording audio locally until the phone comes back into range, as the smartwatch can currently do. The device would also include sensing, allowing researchers to confidently know the wear/non-wear status of the device (either through acceleration data [68, 70, 72] or audio data [4, 5]) to reliably measure and understand adherence/non-response rates in different contexts. Notable is that participants may “wear” the device but not on the ears, such as around the neck.

To be safely deployed in a research study where the device is worn continuously, a prompting device should not obstruct users’ hearing. For some participants, an outside-the-ear device may also maximize comfort. A device deployed for research that is not a person’s personal headset should permit simultaneous use of a personal headset/earpiece. The form factor of the device needs to support different postures, and some people might not prefer a band around the head or neck, especially those with longer hair. A discreet appearance may also increase the likelihood that participants in a client-facing role will wear the prompting device at work. The limitations of the current form factors for head-worn devices contributed to the participants’ positive impression of the smartwatch.

### 6.3 Design Implications for Multimodal Prompting Systems with Real-time Feedback

Researchers have studied the contextual biases related to the response rate in multiple-choice *μ*EMA on the smartwatch [57]. In this study, we explored the factors that affect compliance in a voice-based micro-interaction. Through our preliminary comparison with *μ*EMA, participants also noted scenarios where they would have preferred to switch between voice-based micro-interactions and *μ*EMAs, depending on their contextual and social situation. The findings from this pilot suggest that future systems might maximize participant comfort, satisfaction, compliance, and data quality by combining different prompting modalities (e.g., earpieces, smartwatch, computer) and different reporting mechanisms (e.g., speech, tapping, typing) based on the passively detected contexts of the participants (e.g., location, conversation level, physical activity level) to maximize compliance rate.

Notably, participants in both the usability study and the free-living study mentioned social burden – *not rates of prompting as high as once every five minutes* – as the most significant barrier to the long-term deployment of the self-report system. Participants noted that answering audio-*μ*EMA prompts during a conversation can disrupt the flow of the interaction with others and might be considered “rude” (F1, F3, F9) or create the impression that the participants are not paying attention to the other person. Participants reported that the likelihood of non-response increased when they were interacting with strangers because they did not want to have to explain their behavior, which they found burdensome and stressful. Most participants reported that they had no issues doing the verbal reporting when they were alone – even at a high sampling rate and when they were engaged in cognitive activities. This suggests that one way future high temporal density self-report systems might reduce perceived burden and increase compliance is to employ real-time conversation detection [9, 76] and either decrease the prompting frequency when the users are talking (to minimize interruption burden) or send multiple-choice, text-based *μ*EMA/EMA prompts through a different modality (e.g., the smartwatch). Some participants reported that answering a *μ*EMA question on the smartwatch when interacting with other people was more acceptable than the audio input on the headset.

The environmental context played an important role in how participants felt about the high temporal density self-reporting. Not surprisingly, participants expressed concerns about reporting via voice in public spaces that require low levels of noise, such as libraries and movie theaters – even though they were told they could whisper into the device. The workplace was especially challenging for several participants with client-facing roles; they expressed concerns about being viewed as “rude” (F1, F3, F9) or “unprofessional” (U4, U7, U18, P8) when doing the reporting at work via the headsets. Real-time location awareness might be employed in future systems to automatically change the modality based on both the type of place (e.g., workplace) and the overall noise levels. Our work suggests that some of the participants might prefer *μ*EMA on the smartwatch with the silent and more socially acceptable tapping action while interacting with clients at work, but they will be more inclined to do the verbal reporting that can be accomplished hands-free and possibly at a higher sampling rate when they are alone and walking about or doing computer work.

### 6.4 Feasibility and Challenges for Human-in-the-loop Real-time Recognition Systems

We chose the test domain of providing posture and activity data because recently there has been increasing interest in creating activity recognition systems that can recognize personal activity patterns [22, 24, 50, 53, 56] and continually adapt to changes in human behaviors [1, 2, 28, 36], which requires annotated data of activity and posture while people use wearable sensors. Our study demonstrates the feasibility of collecting temporally dense in-situ behavioral labels using wearable devices via speech. A future system might employ the audio-*μ*EMA method to gather temporally dense data annotations used to train real-time machine learning algorithms [6]. This work suggests that audio-*μ*EMA might be integrated into activity recognition systems that use sensing data from wrist-worn or head-mounted devices [13, 45, 52, 55, 62] to collect in-situ ground truth labels. We have demonstrated that some users can sustain temporally dense activity and posture labeling for a full week at labeling rates of up to twelve times an hour. Additionally, results from our study show the ability of our system to capture a diverse range of background noises, which might be used to increase the accuracy of multimodal HAR models [3, 12, 55].

We qualitatively investigated the quality of the labels collected by examining the consistency and diversity of the activity and posture labels (**RQ2**). A challenge observed in our study is the variability of the responses given by participants. Some participants prefer to report short, ambiguous physical activity labels (e.g., “*sitting and working*”), while other participants tend to give unnecessarily verbose responses (e.g., “*sitting and doing nothing*”). The issue of over-simplistic and unnecessary elaborated speech input has been observed in prior literature on voice-based systems [27, 46]. Ambiguous labels are unhelpful in training activity recognition models, and verbose labels can increase the burden on participants by increasing interaction time. Improving training materials might partially improve the quality of the labels; however, participants might not be able to remember the instructions in real-world settings. Another possible solution is to use real-time processing and ask follow-up questions or provide in-situ feedback from the system when a missing or unintelligible or ambiguous response is detected. Our study suggests, however, that to deploy a real-time feedback system, an accurate automatic speech recognition needs to be integrated into the system, and this itself is a research challenge.

We investigated the audio quality of the recordings collected using our system, and whether an off-the-shelf speech-to-text model can correctly identify the activity and posture labels (**RQ3**). The poor performance of Google speech recognition observed in our study may be attributed to three factors. First, in free-living settings, background noises can affect the quality of the audio and overpower the participants’ voices. Second, the speech recognition model is trained to recognize meaningful commands from the users by leveraging sentence context. However, in our study, participants are trained to report short, unnatural phrases (e.g., “*sitting talking*”), sometimes in the middle of a conversation; this lack of context (or unrelated surrounding context) can confuse the speech recognizer. Third, Google’s open-source speech recognition model appears to be biased towards Google Assistant commands (e.g. “*sitting and watching TV*” is transcribed as “*setting alarm to 4 pm*”). The 78.2% gap between human annotation and automatic annotation suggests the need for future research on improving transcription accuracy by adapting or fine-tuning the model to favor postures and physical activity keywords (e.g., when speech-to-text encounters the word “*sitting*,” it should transcribe the word as “*sitting*” more often than “*setting*”). Google Cloud Speech-to-text Service also offers features to improve transcription accuracy in noisy environments by supplying audio clips with noise or expanding the vocabulary of the model using custom audio files (e.g., “*mounting the computer*” was not present in the open-source version of Google speech-to-text model, but this is an activity label observed in our study from a participant who was moving apartments).

One value of real-time feedback is that it could be used to train participants out of behaviors that make self-reporting more difficult than it needs to be. We found that some participants felt the need to bring their hands close to their mouths in the smartwatch audio-*μ*EMA condition because they assumed that would be the only way to provide quality audio. This arm movement, however, negates the advantages of audio-*μ*EMA on the smartwatch, because reporting is no longer hands-free, which at a rate of once every five minutes, contributes to the burden. It also makes *μ*EMA less effective during activities that require the use of hands. Real-time feedback [48] might reduce participants’ uncertainty with the audio quality of the devices and thus allow them to learn that they can speak without changing the hand position (which we have shown can lead to reasonable quality audio). However, this real-time solution is difficult to implement given the current quality of the speech recognition model. Additionally, asking follow-up questions will increase interaction time, making it less likely a prompt and response would be a true micro-interaction, thereby increasing the perceived burden.

## 7 LIMITATIONS AND FUTURE WORK

This was exploratory work, and it has several limitations. First is the small sample size and short study duration, which proved sufficient for identifying challenges to explore in future work but did not allow for longitudinal comparisons.

Another limitation is that our study population is skewed towards young, emerging adults who are generally facile with technology, used to mobile devices that interrupt them frequently, and frequently wearing earbuds. Future work should explore the reactions of other demographic groups.

A third limitation resulted from the need to distribute research equipment. Long-term, audio-*μ*EMA might be deployed using the phones, smartwatches, and/or earables that people already have, but participants in the feasibility study were given a headset, and it may not have been their top choice. Charging and maintaining an extra phone, watch, and headset for up to 21 days is burdensome and, independent of the self-report method, would affect adherence rates. Compliance was impacted when traveling participants forgot to bring the watch/headset charger with them and did not change the time zone on the research phone.

In this pilot study, we checked in with participants daily to remind them (1) of which device to use given their day in study, (2) to answer the end-of-day survey, and (3) to charge all devices at night. We also transcribed the audio recordings from the prior day and if an unusual amount of missing data or unintelligible data were identified, we would inquire about the participant’s behavior. These daily check-in calls built rapport between the researchers and the participants, which can increase response rate and engagement with the system, but they were necessary given the exploratory nature of the study and limited equipment availability. Future studies employing audio-*μ*EMA could automate these reminder tasks using phone notifications or automatic text messages, as well as employ a fine-tuned speech recognition system to automatically detect poor self-report labels from participants and immediately provide feedback.

Finally, the main purpose of this pilot study was to explore the feasibility of audio-*μ*EMA on various devices to inform and motivate future studies. We used the domain of reporting posture and activity to test the concept. However, the method could also be used to gather other types of self-report information, and future studies should explore domains such as measurement of mood and stress and ascertain if the nature of the information being reported impacts the viability of the method.

## 8 CONCLUSION

In this paper, we present a novel data collection method, audio-*μ*EMA, using a new speech-based microinteraction self-report – where participants are prompted via a short *beep* through a headset or a short vibration on the wrist to report their behavioral data. We conducted a one-hour usability study and a within-subject 6–day to 21-day free-living study and examined the feasibility of an in-the-wild deployment of our system. Despite the temporal density of the prompts, participants were highly engaged with our system, with an average completion rate of 67.7% and a compliance rate of 60.9%. We considered the usefulness of the labels recorded from the feasibility study and investigated the quality of the audio recordings collected using our system. Our usability study and field deployment shows the potential of leveraging audio-*μ*EMA for gathering rich, real-time behavioral data, whether as a standalone data collection system or integrated within a real-time activity recognition framework.

## Supplementary Material

audio clips for the audio quality experiments

## Figures and Tables

**Table 1:** Summary of audio-input systems used in closely related research.

Prior work	Modality	Data Collected	Response Type	Prompting Interval	Prompting Period	Interaction Duration	Study Duration
Kouba et al. [43]	mobile phone	speech biomarker of Parkinson’s disease	open-ended	1/day and self-invoked	N/A	N/A	2 years
Doherty et al. [26]	mobile phone	human activity & wellbeing	open-ended	1/35 min	8AM – 10PM	2–79 s	1 day
Wei et al. [77]	smart speaker	availability, mood, boredom & current activity	number & open-ended	1/60 min	9AM - 10PM	40 s	3 weeks
Chen et al. [21]	smart speaker	quality of sleep, social interaction, exercise, pain management, alcohol uses, food consumption, medication management	binary, Likert, number & open-ended	1/day	end-of-day	≤5 min	40 days
Maharjan et al. [49]	smart speaker	mental health & wellbeing	open-ended dialogue	self-invoked	N/A	N/A	4 weeks
Kim et al. [40]	smartwatch	physical activity, duration & perceived effort level	open-ended	1/30 min and self-invoked	when participants wear the watch	2 min	7 days
Hester et al. [32]	smartwatch	word-retrieval responses	open-ended	1/15 min	10 AM – 8 PM	10 s	21 days
Bi et al. [16]	earbuds	pain, exertion, desire to stop, emotional valence in runners	number & ordinal scale	1/5 min	30–60 min per session	N/A	30–60 min
Bi et al. [17]	earbuds	pain, worry, confidence in task	number & ordinal scale	1/min	10–30 min per session	N/A	10–30 min
Jang et al. [35]	frame-shaped monitor	daily emotions	open-ended	self-invoked	N/A	50–110 s	3 weeks
Silva and Epstein [48]	mobile phone and smartwatch	food consumption	open-ended	self-invoked	N/A	unknown	2 weeks
Lou et al.[46]	mobile phone	food consumption	guided responses	4/day	After each meal and EOD	*M*=149 s, *SD* = 97.31	7 days
Lou et al. [47]	mobile phone	exercise	open-ended	self-invoked	N/A	unknown	2 weeks
**audio-*μ*EMA**	**earables, smartwatch**	**postures & physical activity**	**open-ended**	**1/ 2–5 min**	**waking day**	**7–10 s**	**7 days**

**Table 2:** Scenarios in the usability study protocol.

Scenario	Potential Postures	Potential Physical Activities	Environment	Expected Intensity
Verbally answer demographics questionnaire	sitting	talking/having conversation	quiet office, one-on-one conversation	sedentary
Go on a tour of the building	upright/standing	talking/going on a tour/walking/riding the elevator	open office, public area	light
Do a crossword puzzle	sitting	writing/doing a crossword puzzle/reading	quiet office, alone	sedentary

**Table 3:** Summary of participant demographics.

Variable	Participants from usability study (n = 18)	Participants from feasibility study (n = 15)
Age (years)	M = 22.94, SD = 3.24, range = 19–24	M = 21.9, SD = 2.8, range = 18–29
Gender	8 Male, 10 Female	8 Female, 7 Male
Race	10 Asian, 2 Black, 6 Caucasian	11 Asian, 1 Black, 3 Caucasian
Technical Proficiency*	12 Somewhat Comfortable, 6 Extremely Comfortable	8 Somewhat Comfortable, 7 Extremely Comfortable
Physical Activity Level**	11 Sedentary, 3 Moderate, 4 Active	7 Sedentary, 6 Moderate, 2 Active
Occupation	7 Undergraduate Students, 5 Graduate Students, 2 Clinicians, 2 Personal Trainers, 2 Office Workers	2 Undergraduate Students, 7 Graduate Students, 1 Part-time Cashier, 1 Clinician, 1 Office Worker

* Response to the question “*Are you comfortable using new technology or software that you haven’t encountered before?*”

** Categorized based on the responses to the questions “*How much time on a typical day do you spend doing moderate/vigorous physical activity?*”

**Table 4:** Overall completion and compliance rates (%), and the number of audio and multiple-choice (MC) responses for participants across the three study durations and conditions.

Metrics	Study Duration (days)	*μ*EMA	Audio- *μ*EMA (headset)	Audio- *μ*EMA (watch)
Completion (%)	2	80.37	73.9	72.2
	4	66.8	64.1	67.6
	7	73.9	65.7	68.5
Compliance (%)	2	51.2	58.6	56
	4	57.2	61.2	59.9
	7	56.6	52.5	55.9
Total number of answered prompts	2	172	565	658
	4	237	712	853
	7	2,061	5,738	6,106

**Table 5:** Captured postures, high-level physical activity, and context labels from the participants’ verbal self-reports.

Labels	Label	Count	Variations	Example Variations
Posture	Sit	4966	23	“sitting down”, “sitting upright”, “sit”, …
	Stand/upright	2623	15	“standing”, “standing upright”, “upright”, “standing up”, …
	Lie	1528	8	“lying on my back”, “lying on my side”, “lying on my stomach”
	Bend over	95	7	“bending down”, “bending over”, “bending”, …
	Recline	56	2	“reclining”, “recline”
	Lean	51	7	“leaning over”, “leaning back”, “leaning against”, …
	Crouch	13	3	“crouching”, “crouch”, “slouch”
	Kneel	9	2	“kneeling”, “kneel”
Activity	Screen time	911	69	“read an email”, “on my computer”, “on phone”, “watch TV”, …
	Commute	784	59	“walking down the street”, “walking outside”, “cycling”, “riding a scooter”, “waiting for the bus”, …
	Social	674	74	“facetime”, “talking”, “talking to my friends”, “on the phone call”
	(School) Work	568	47	“doing homework”, “fixing a bug”, “working on a project”, …
	Cook/Eat/Drink	521	88	“cutting tomatoes”, “eating cereal”, “making snack”, “drink water”
	Chores	496	91	“cleaning”, “doing the bed”, “doing laundry”, “cleaning my kitchen”
	Self-maintenance	493	31	“getting ready for the shower”, “applying makeup”, “changing clothes”, “taking my meds”, “brushing hair”
	Hobby/Leisure	438	44	“reading manga”, “playing games”, “build Lego”, “doing cross stitches”
	Exercise	44	13	“doing exercise”, “stretching”, “walking on treadmill”, …
	Others	659	42	“open the door”, “adjusting my headphone”
Context	Location	4013	229	“outside”, “indoors”, “in my apartment”, “at work”, “on my chair”, “on my bed”, “in a restaurant”, “in a class”
	Device	1090	54	“on my laptop”, “on my phone”, “on my computer”
	Transportation	315	29	“electric scooter”, “on the train”, “on the bus”, “in the taxi”

**Table 6:** Percentage of different types of background noises presented in all headset/watch audio recordings from most common to least common. Percentages are calculated over all headset and smartwatch recordings.

Background noise	Descriptions/Examples	% (count)	
		Headset	Watch
conversation	background conversation or participant’s conversation	37.9 (4,020)	43.8 (4,812)
TV	sound coming from electronic devices (TV, computer, smartphone)	18.6 (1,973)	8.2 (900)
music	melodious, musical sound	9.3 (986)	7.8 (338)
weather	sound made by weather-related source (rain, wind)	8.0 (215)	6.9 (747)
water	sound coming from water (washing hands, cooking)	1.3 (139)	1.4 (154)
human touch	sound made by human actions (slam doors, walk up/downstairs, putting on clothes)	1.3 (138)	1.8 (195)
mechanical	periodic sound made by non-human/animal objects	1.2 (131)	0.9 (99)
conductor	sound made by speaker on the bus or train	0.4 (43)	0.02 (22)
traffic	sound made by traffic (car honk, engine sound)	0.2 (23)	1.1 (120)
ringtone	periodic musical sound coming from electronic devices	0.2 (20)	0.02 (25)
animal	sound made by animals (dog barking)	0.06 (6)	0.09 (10)
unknown	unidentifiable source of background noise	7.8 (827)	5.7 (622)

**Table 7:** Accuracy of Google’s open-source speech recognition on intelligible recordings.

Labels type	Audio clips with background noises		Audio clips without background noises		Total	
	watch	headset	watch	headset	watch	headset
Posture	23%	19.3%	26.1%	23.3%	24.2%	20.8%
Activity/context	24.5%	16.2%	25.5%	24%	24.7%	19.2%

**Figure 5:**
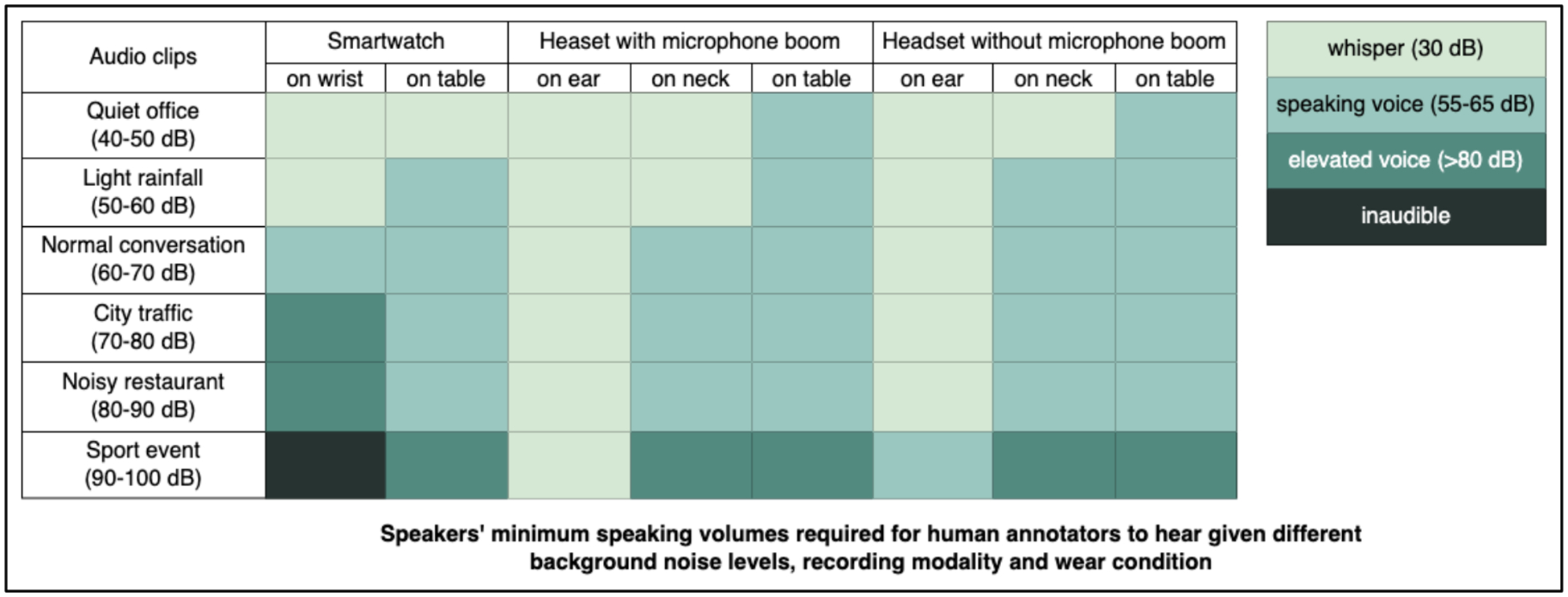
Results from the controlled audio quality experiment.

## Box

CCS CONCEPTS • **Human-centered computing** • **Ubiquitous and mobile computing** • **Empirical studies in ubiquitous and mobile computing**

## Footnotes

1
https://pypi.org/project/SpeechRecognition/

## APPENDIX A: AUDIO QUALITY ACROSS MODALITIES

We present the trade-offs between different implementations for audio-*μ*EMA discussed above and in the remainder of the paper in Table 8. These trade-offs are compiled from participants’ feedback in the usability studies, as well as common sense knowledge.

**Table 8:** Trade-offs between different recording modalities for audio-*μ*EMA.

Prompting Modality	Advantages	Disadvantages
Bone-conduction headset with microphone boom (OpenComm)	Good audio quality	Custom charging cable
	Battery lasts for three days*	Heavier compared to other options
	Can be worn around the neck	Mic boom visually noticeable
	Can be worn with personal earbuds	Needs Bluetooth connection to phone
	Sends persistent notifications when user goes out of range with the phone	Needs to be manually turned on and reconnected to the phone if phone goes out of Bluetooth range
Bone-conduction headset without microphone boom (OpenRun)	Good audio quality	Custom charging cable
	Battery last for two days *	Needs Bluetooth connection to phone
	Lightweight	Does not notify user when being
	Aesthetically common	disconnected to the phone
	Can be worn with personal earbuds	Needs to be manually turned on and reconnected to the phone if phone goes out of Bluetooth range
Open-ear wireless earbud (OpenFit)	Good audio quality	Battery only lasts 8 hours*
	Lightweight	Easy to misplace
	Aesthetically common	Uncomfortable to wear on top of personal earbuds
	Automatically turns on and connects to the phone	Needs Bluetooth connection to a phone
	Can be worn in one ear	Does not notify user when disconnected
	Widely used USB C charging cable	with the phone
Smartwatch (Fossil Gen 5/6)	Lightweight	Poor audio quality
	Aesthetically common	Unpredictable and poor battery life (10–15 hours per charge)
	No accompanying phone required	Custom charging cable

* Battery was measured while headset is connected to phone, and prompting was turned on (7 s of recording once every 5 min)

We conducted a controlled lab measurement to explore the best-case audio quality we might expect to obtain from the three different audio-*μ*EMA modalities (i.e., Fossil smartwatch, OpenComm headset, OpenRun headset) under different levels of background noise and wear conditions. We tested five different wear and use conditions: headset-on-ear, headset-around-neck, headset-on-table, watch-on–wrist, and watch-on-table. Three non-native English speakers (two females, and one male) repeated the same phrase: “sitting in a restaurant” at three different speaking volumes: whisper (approximately 30 dB), normal speaking voice (approximately 55–65 dB), and elevated voice (>80 dB). We chose six audio clips of real-world scenarios and adjusted the noise levels according to Figure 2. We measured and set the background noise level using a Tadeto digital sound meter (model TE017) placed about 15 cm from the headset and the smartwatch. Two non-native English-speaking researchers listened to the audio clips recorded and marked the intelligibility of the verbal responses. An audio clip was considered “intelligible” if both annotators independently indicated that they could hear the phrase.

Figure 5 shows the minimum volume speakers needed for both annotators to confidently recognize the speaker’s speech in each of the different wear conditions with different background noise levels. In both the headset-on-table and smartwatch-on-table conditions, the devices are placed 30 cm away from the speaker’s mouth. In the watch-on-wrist condition, the speaker was standing upright with their hand parallel to their body at their side. For each entry in the figure, we report the intelligibility of the audio clip with the lowest rating from the three speakers. We decided to use the poorly rated audio clip to cover all edge cases (e.g., when a participant has a particularly quiet voice).

Notably, the annotators in this test were aware of the phrase being spoken, which would bias these results towards recognition. These results are therefore best-case. As we discuss in subsequent sections, sometimes participants wore the headsets around their necks, which is why we tested that condition.

Overall, the headsets worn properly on the ears appeared to have better audio quality than the smartwatch. However, the difference between the audio intelligibility of the headset with the microphone boom (OpenComm) and without (OpenRun) across different wear conditions was modest.

## APPENDIX B: MATERIALS FOR TRAINING SESSIONS

**Table 9:** Lists of example postures and physical activities presented to the participants. They were told their response to a prompt for posture and activity did not have to be a full sentence (i.e., “*I’m sitting and, in a conversation*,” and “*sitting, talking*” are both acceptable responses), and if the physical activity implied posture, participants only needed to report the physical activity (“*upright and walking indoors*” and “*walking indoors*” are both acceptable responses).

Postures	Physical Activities
Upright	Type on the computer
Sit	Cook dinner
Lie	Doing paperwork
Kneel	Watering plants
Crouch	Chopping vegetables
Bend over	Stand on the train
Recline	Having conversation
Jump	Run on treadmill
Stand	Drink water
	Doing chores
	Watch videos on phone/computer
